# Cardiac coherence and medical hypnosis: a feasibility study of a new combined approach for managing preoperative anxiety in patients with breast or gynaecological cancer

**DOI:** 10.1016/j.bjao.2024.100309

**Published:** 2024-09-24

**Authors:** Jibba Amraoui, Gilles Leclerc, Marta Jarlier, Jesus Diaz, Ridvan Guler, Clément Demoly, Catherine Verin, Sophie Rey Dit Guzer, Patrick Chalbos, Aurore Moussion, Christophe Taoum, Mathias Neron, Laurent Philibert

**Affiliations:** 1 Department of Anaesthesia, Montpellier Cancer Institute, University of Montpellier, Montpellier, France; 2 Biometrics Unit, Montpellier Cancer Institute, University of Montpellier, Montpellier, France; 3 Department of Clinical Research and Innovation, Montpellier Cancer Institute, University of Montpellier, Montpellier, France; 4 Department of Surgical Oncology, Montpellier Cancer Institute, University of Montpellier, Montpellier, France; 5 Department of Pharmacy, Montpellier Cancer Institute, University of Montpellier, Montpellier, France; 6 INSERM U1194, Montpellier Cancer Research Institute (IRCM), University of Montpellier, Montpellier, France

**Keywords:** cardiac coherence, feasibility study, medical hypnosis, non-pharmaceutical approach, preoperative anxiety

## Abstract

**Background:**

Non-pharmaceutical approaches can help manage preoperative anxiety, but few studies have evaluated psychoeducational programmes, especially for cancer surgery. We assessed the feasibility of the COHErence Cardiaque (COHEC) programme where cardiac coherence and medical hypnosis are combined to manage preoperative anxiety in patients undergoing breast or gynaecological cancer surgical interventions (BGCSI).

**Methods:**

Patients undergoing BGCSI were enrolled and followed a daily home programme with cardiac coherence and medical hypnosis sessions, starting 7 days before the procedure. The primary endpoint was optimal patient adherence (i.e. completion of ≥14 sessions). Secondary endpoints were anxiety levels, measured using the Visual Analogue Scale (VAS) and the Amsterdam Preoperative Anxiety and Information Scale (APAIS), satisfaction (EVAN-G), and quality of postoperative recovery (QoR-15).

**Results:**

In total, 53 patients [mean age: 55 (34–82) yr] were included; 83.7% had breast cancer and 15.1% had gynaecological cancer. Optimal adherence was achieved by 64.2% (95% confidence interval: 49.8–76.9%) of the intention-to-treat population. Among the 43 patients who completed at least one session, exploratory analysis showed that anxiety on the day before (*P*=0.02) and the morning of the intervention (*P*=0.04) was decreased in patients with severe anxiety at baseline (VAS ≥70). The median VAS satisfaction score for the programme was 10 (4–10). Overall, 94% of patients were willing to include the COHEC programme in their daily routine.

**Conclusions:**

The implementation of a psychoeducational programme combining cardiac coherence and medical hypnosis is feasible and might potentially help patients undergoing BGCSI to manage preoperative anxiety. A randomised trial is underway to assess the efficacy of the COHEC programme.

**Clinical trial registration:**

NCT03981731.

Preoperative anxiety affects approximately 50% of patients and is associated with significant morbidity, higher surgical complication rates, and patient dissatisfaction with surgical outcomes.^1^^,^^2^ Benzodiazepines, especially midazolam,^3^ were commonly used to manage preoperative anxiety; however, they are now used less frequently with the increasing adoption of day surgery.^4^ Moreover, they are associated with adverse effects, particularly in older adults.^5^ Non-pharmacological approaches, such as medical hypnosis, music therapy, and cognitive-behavioural techniques, have shown promising results.^6^ However, their implementation relies on the healthcare team's willingness, training, and investment. Their use can also be challenging in highly distressed patients, including patients with cancer. New approaches (e.g. cognitive-behavioural programmes) have shown real benefits, but they are not suitable for patients undergoing interventions for cancer removal because they require long-term supervised practice.^7^ Therefore, alternatives are needed.

The mechanisms underlying anxiety have been extensively studied.^8^ Several structures are involved, including the limbic system, autonomic nervous system, immune system, and hypothalamic–pituitary–adrenal axis. Various mediators, mainly controlled by the autonomic nervous system, mediate the interactions between limbic system and brainstem, hypothalamus, and prefrontal cortex. In stressful conditions, they induce structural and functional changes in the neural architecture and modify synaptic activity. Among the many factors that influence stress levels, some can be modulated (e.g. mental schemas, perceived resources, perceived control, and perceived stress) and others cannot (e.g. patient characteristics, biological state, and individual cognition).^8^^,^^9^ As anxiety pathophysiology and management are complex, a multimodal patient-tailored approach should be used. We propose to combine two approaches: medical hypnosis and an innovative breathing technique called cardiac coherence. Medical hypnosis acts on the central nervous system, especially the limbic system, whereas cardiac coherence acts on autonomic nervous system regulation. Several studies demonstrated their benefits in managing pain and anxiety.^10^, ^11^, ^12^, ^13^ Cardiac coherence involves guided breathing (6 bpm) and leads to synchronisation of the brain structures involved in management of emotions. The combination of cardiac coherence and medical hypnosis may enhance and potentiate their positive effects on anxiety. In addition, patients could use these non-pharmacological approaches whenever they want throughout the cancer care pathway, with minimal side effects.

The aim of the COHErence Cardiaque (COHEC) study was to assess the feasibility of a psychoeducational programme that combines cardiac coherence and medical hypnosis, starting 7 days before the intervention to manage anxiety in patients undergoing breast or gynaecological cancer surgical interventions (BGCSI).

## Methods

### Study design and participants

This prospective, single-centre study (NCT03981731) was conducted at Montpellier Cancer Institute, Montpellier, France, in accordance with the French Public Health Code, Good Clinical Practice, and Declaration of Helsinki. An Ethical Committee (Comité de Protection des Personnes Nord Ouest III, accepted on 30 October 2019) approved the study.

Inclusion criteria were patients ≥18 yr undergoing planned BGCSI that would require a hospital stay ≤3 days, signature of the informed consent form, and coverage by the French social security system. Exclusion criteria were outpatient surgery, bradycardia (<50 beats min^−1^) or arrhythmia, β-blocker treatment, and severe heart failure (ventricular ejection fraction <40%).

### Objectives

The primary objective was to evaluate the COHEC programme feasibility.

Secondary objectives were (1) to assess the anxiety levels at inclusion the day before (D–1) and the day of the surgical intervention (D0), and (2) to assess the patients' perioperative experience, recovery, and satisfaction.

### Outcome measures

The primary outcome was the optimal adherence rate to the COHEC programme. Patients were categorised as a function of their adherence level: optimal (≥67% of sessions, ≥14/21 sessions), satisfactory (≥50% and <67%, 11 to13/21 sessions), moderate (≥33% and <50%, 7 to 10/21 sessions), and poor (<33%, <7/21 sessions) adherence. Secondary outcomes included were: recruitment rate (proportion of eligible patients who agreed to participate) and reasons for refusal; Visual Analogue Scale (VAS) scores (0–100, 0: no problem) to assess anxiety and satisfaction; Amsterdam Preoperative Anxiety and Information Scale (APAIS) scores; ‘Evaluation du Vécu de l'Anesthésie Générale’ (EVAN-G) questionnaire score (French questionnaire to assess the perioperative experience concerning anaesthesia); 15-item Quality of postoperative Recovery questionnaire (QoR-15) score; percentage of patients planning to use the technique after the study and percentage of patients willing to recommend the technique. Table 1 summarises the data collection schedule.

**Table 1:** Data collection schedule. APAIS, Amsterdam Preoperative Anxiety and Information Scale; D–1, day before the intervention; D0, day of the intervention; D1 and D2, day 1 and 2 after the intervention; EVAN-G, ‘Evaluation du Vécu de l'Anesthésie Générale’ (EVAN-G) questionnaire score (French questionnaire to assess the perioperative experience concerning anaesthesia); QoR-15, 15-item Quality of postoperative Recovery questionnaire score; VAS, Visual Analogue Scale. ∗Including age, sex, and clinical (disease history, anxiolytic and antidepressant treatments) data. ^†^Measured before and after the first cardiac coherence session. ^‡^Sessions recorded by the patients using the monitoring form.

	Preanaesthesia consultation	D–1	D0	D1	D2
Participants' baseline characteristics∗	✓				
Heart rate variability measurement	✓^†^	✓			
ASA score	✓				
Cardiac coherence and medical hypnosis sessions^‡^	✓	✓	✓		
APAIS	✓	✓			
EVAN-G				✓	✓
QoR-15	✓			✓	✓
VAS anxiety	✓	✓	✓	✓	
VAS satisfaction				✓	

### The COHEC programme

Before the study, clinical research assistants (CRAs) received 1.5 h of training in cardiac coherence practice and emWave® Pro use (HeartMath, Inc. Quantum Intech, Boulder Creek, CA, USA, www.heartmath.com).^14^ Patients followed a 7-day home programme that combined medical hypnosis and cardiac coherence sessions. The ‘365’-based cardiac coherence session consisted of six breathing cycles min^−1^ for 5 min,^15^ using the free RespiRelax® application (Les Thermes d’Allevard, Allevard, France). Each cycle comprised 5 s of inhalation and 5 s of exhalation. The 8-min Ericksonian-based medical hypnosis listening session was developed by an anaesthetist with expertise in medical hypnosis to help patients relax by focusing on their breathing. Patients were asked to do the cardiac coherence session three times per day before meals, according to the 365 method.^15^ They could do the medical hypnosis session independently or in combination with the cardiac coherence session. Patients recorded all cardiac coherence and medical hypnosis sessions in the monitoring form.

### Cardiac coherence state evaluation

Heart rate variability (HRV) was recorded for 5 min at baseline and at D–1 using a plethysmograph and analysed using the emWave Pro® software. Fourier analysis was used to obtain a power spectral density *vs* frequency plot as previously described.^16^ Cardiac coherence was achieved when a peak at 0.1 Hz was observed on the plot (qualitative evaluation), corresponding to six breathing cycles min^−1^.

### Procedure

At inclusion, the investigator detailed the care process, recorded the patients' baseline characteristics, and provided the monitoring form. A trained CRA explained the cardiac coherence method, performed a session with the patient, and monitored HRV before and after the training session to illustrate the effect of cardiac coherence, to help the patient achieve and maintain a state of coherence, and to confirm that the patient had mastered the technique. A link to the medical hypnosis tape session was e-mailed to the patient. Patients were asked to perform the COHEC programme at home for 7 days. At D–1, the CRA recorded the HRV. At D0, the patient listened to the medical hypnosis tape and performed the cardiac coherence session in the morning before the procedure. The anaesthetist performed a preoxygenation session, and asked the patient to use the cardiac coherence technique while listening to the medical hypnosis session. The anaesthetic protocol was left to the anaesthetist's choice, based on the nature and complexity of the intervention, and included propofol, opioids (sufentanil), and neuromuscular blocking agents (cisatracurium besilate, rocuronium bromide), according to institutional and good practice guidelines.

### Sample size calculation

The sample size calculation was based on the adherence to the COHEC programme. The programme consisted of 21 sessions, and optimal adherence was considered to be achieved when patients completed at least 14 cardiac coherence sessions in the 7 days before the surgical intervention. To have ∼75% of patients with optimal adherence to the programme, with a 95% confidence interval (CI), 44 patients were required.^17^ Considering 20% of patients as non-evaluable, 53 patients needed to be included.

### Statistical analysis

Qualitative variables were described using frequencies and percentages and quantitative variables using mean (standard deviation [sd]), median, and range. The percentage of patients who adhered optimally to the programme was presented with its 95% CI, and the adherence level distribution was reported. The VAS, APAIS, EVAN-G, and QoR-15 scores were presented as mean (sd). VAS scores (range 0–100) were classified as low (≤30/100), moderate (30–70), or severe (≥70/100). The APAIS included a total anxiety score, and anxiety sub-scores related to fear of anaesthesia, fear of intervention, and need of information.^18^ The EVAN-G questionnaire included a global index and six specific sub-scores on attention, privacy, information, pain, discomfort, and waiting time.^19^ The QoR-15 scale scores were reported as continuous variables.^20^ Scores between time points (exploratory analyses) were compared with the Wilcoxon matched-pairs signed-rank test, and categorical variables with χ^2^ test. Baselines characteristics, baseline scores, and feasibility (i.e. primary outcome, assessed by the optimal adherence proportion) were analysed on the intention-to-treat population (study withdrawals being considered as non-adherence). Exploratory analyses (anxiety, scores over time) were analysed in patients who did at least one session of the programme (per-protocol population).

The statistical significance level was set at *P*<0.05. Analyses were performed with the Stata® v.16 software (StataCorp LP, College Station, TX, USA).

## Results

### Study population

**Fig. 1:**
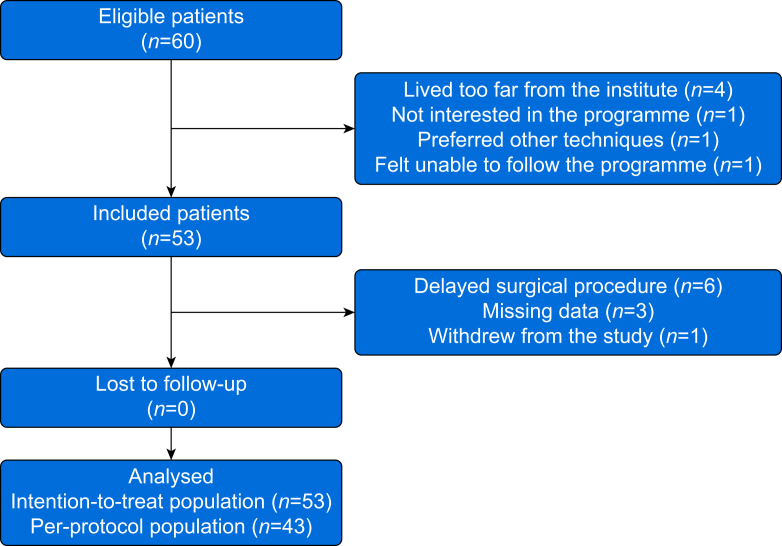
Flow chart.

Fifty-three patients (intention-to treat population) were enrolled between February 2020 and January 2022 (flowchart in Fig 1). The recruitment rate was 88.3% (95% CI 77.4–95.2%). Their characteristics at inclusion are in Table 2.

**Table 2:** Patients' baseline characteristics. Data are presented as *n* (%) or median (range). ∗Mastectomy with or without reconstruction.

Variable	
**Age** (yr)	55 (34–82)
**Heart rate** (beats min^−1^)	74 (60–100)
**Body mass index** (kg m^−2^)	23.1 (15.63–48.45)
**Tobacco consumption**	
No	32 (83.0)
Yes	9 (17.0)
**ASA score**	
1	14 (26.4)
2	37 (69.8)
3	2 (3.8)
**History of general anaesthesia**	
No	4 (7.5)
Yes	49 (92.5)
**If yes, memories of waking quality**	
Good	32 (65.3)
Poor	11 (22.4)
Do not know	6 (12.2)
**Cancer site**	
Gynaecological (ovary, uterus)	8 (15.1)
Breast∗	45 (84.9)

### COHEC programme feasibility

Adherence to the COHEC programme was optimal in 64.2% (95% CI 49.8–76.9%) of patients in the intention-to-treat population (*n*=53; Table 3), and only 3.8% (*n*=2/53) of patients completed fewer than seven sessions. In addition, optimal adherence was 79.1% (95% CI 64.0–90.0%) when considering only patients who performed at least one session (*n*=43). The median number of sessions completed was 18 (5–21); 40% of patients listened to the medical hypnosis tape daily and 37% about three times per day.

**Table 3:** Adherence to the COHErence Cardiaque (COHEC) programme. CI, confidence interval; ITT, intention-to-treat; PP, per-protocol.

	*n* (%)	95% CI
**Adherence in the ITT population (*N* = 53)**		
Yes	34 (64.2)	49.8–76.9
No	19 (35.8)	–
**Adherence level in the ITT population (*N*** = **53)**		
Optimal (≥14 sessions)	34 (64.2)	**–**
Satisfactory (11–13 sessions)	3 (5.6)	**–**
Moderate (7–10 sessions)	4 (7.5)	**–**
Poor (<7 sessions)	2 (3.8)	**–**
No session	10 (18.9)	**–**
**Adherence in the PP population (*n*** = **43)**		
Yes	34 (79.1)	64.0–90.0
No	9 (20.9)	**–**

The programme was performed by 86% of patients (per-protocol population) before going to the operating theatre, among whom 10 (25.6%) had severe anxiety (VAS score >70). The other patients said that they did not have enough time. In 94.9% of patients (per-protocol population), anaesthetic induction was performed while performing the COHEC programme. HRV monitoring showed that 51/53 patients (96.2%) had a peak at 0.1 Hz at inclusion and 39/43 patients (90.7%, per-protocol population) at D–1. Four patients (9.3%) had no peak or cardiac coherence at D–1.

### Exploratory analyses of anxiety

**Fig. 2:**
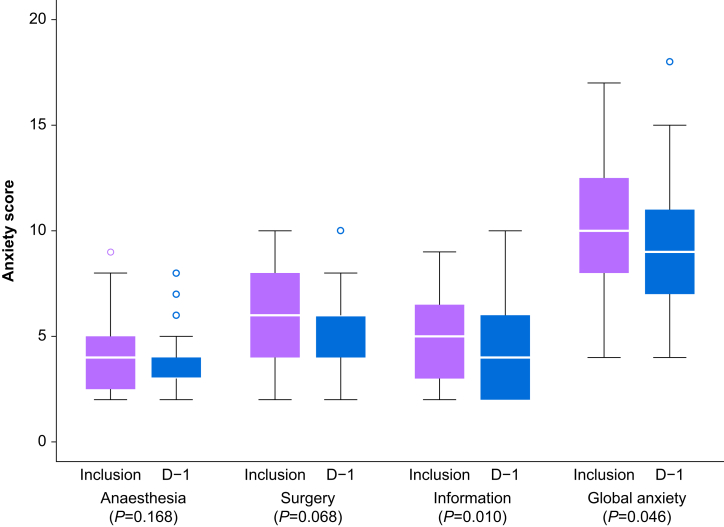
The Amsterdam Preoperative Anxiety and Information Scale (APAIS) scores at inclusion and the day before the intervention (D–1). Changes in anxiety scores (according to APAIS) from inclusion to day before surgery (D–1). Boxplots display the median, the 25th and 75th percentiles, extreme values (whiskers), and aberrant values (hollow circles). *P*-values are based on the Wilcoxon matched-pairs signed-rank test.

The evolution of anxiety was examined. At inclusion, the median VAS anxiety and APAIS total anxiety scores were 40 (0–100) and 10 (4–17), respectively. The VAS anxiety scores did not differ between inclusion and D–1 (*P*=0.23) and between inclusion and D0 (*P*=0.42, paired samples Wilcoxon test). However, anxiety was significantly decreased at D–1 (*P*=0.02) and D0 (*P*=0.04) in the 10 patients with severe anxiety (VAS score ≥70) at baseline. Between baseline and D–1, the APAIS sub-scores for need for information (*P*=0.01) and the APAIS total anxiety score (*P*=0.05) were significantly decreased (Fig 2). Conversely, the scores for surgery-related anxiety (*P*=0.07) and anaesthesia-related anxiety (*P*=0.17) remained unchanged (Fig 2).

The correlation between VAS and APAIS was also explored. The median VAS anxiety score was lower in patients with an APAIS total score <11 than in those with score ≥11, both at inclusion (20 *vs* 65; *P*<0.01, Kruskal–Wallis test) and at D–1 (30 *vs* 65; *P*<0.01).

### Quality of care and patient satisfaction

The median VAS satisfaction score for the COHEC programme training was 10 (4–10). Table 4 summarises the results of the QoR-15 and EVAN-G questionnaires. The QoR-15 results suggested that patients seemed to have a good recovery with a rapid decrease of pain, nausea, vomiting, and anxiety. The mean EVAN-G global index was 73.7 (sd 14.4) at D1 and 71.7 (14.9) at D2.

**Table 4:** Quality of recovery and patient satisfaction. Data are presented as mean (standard deviation). QoR-15, 15-item Quality of postoperative Recovery questionnaire; EVAN-G, Evaluation du Vécu de l'Anesthésie Générale questionnaire (French questionnaire to assess the perioperative experience concerning anaesthesia). Quality of recovery was evaluated using the QoR-15 scale and patient satisfaction was evaluated with the EVAN-G scale. ∗Comparison of QoR-15 total score between inclusion and day 1 (D1) post-intervention (*P*=0.7), D1 and D2 post-intervention (*P*=0.07), and inclusion and D2 (*P*=0.04). ^†^Comparison of the EVAN-G global index between D1 and D2 (*P*=0.12).

	Inclusion	D1	D2
**QoR-15 items**			
1. Able to breathe easily	6.7 (2.4)	8.1 (2.1)	8.6 (1.3)
2. Been able to enjoy food	7.1 (2.1)	7.4 (2.5)	8.1 (1.7)
3. Feeling rested	5.4 (2.1)	6.5 (2.2)	6.9 (2.1)
4. Have had a good sleep	5.6 (3.0)	4.9 (2.7)	6.3 (2.3)
5. Able to look after personal toilet and hygiene unaided	9.9 (0.6)	7.7 (2.4)	8.0 (2.1)
6. Able to communicate with family or friends	9.1 (1.8)	9.0 (1.8)	9.2 (1.2)
7. Getting support from hospital doctors and nurses	2.6 (3.6)	5.0 (2.7)	3.6 (3.2)
8. Able to return to work or usual home activities	8.6 (2.6)	5.9 (2.4)	6.5 (2.6)
9. Feeling comfortable and in control	7.8 (2.1)	6.9 (2.2)	7.5 (2.2)
10. Having a feeling of general wellbeing	7.0 (2.3)	6.6 (2.2)	7.0 (2.1)
11. Moderate pain	4.4 (3.0)	4.6 (2.7)	4.1 (2.8)
12. Severe pain	2.6 (3.1)	2.1 (2.4)	1.6 (2.5)
13. Nausea or vomiting	1.3 (2.5)	1.2 (2.4)	0.5 (1.8)
14. Feeling worried or anxious	5.2 (3.0)	2.6 (2.6)	2.2 (2.7)
15. Feeling sad or depressed	3.4 (3.3)	1.7 (2.2)	1.7 (2.5)
Total score∗	94.1 (16.5)	96.0 (17.3)	101.3 (17.0)
**EVAN-G dimensions**			
Attention	**–**	84.9 (14.8)	83.4 (13.7)
Privacy	**–**	76.2 (19.2)	76.4 (18.5)
Information	**–**	70.1 (21.3)	68.7 (20.8)
Pain	**–**	66.9 (25.3)	62.8 (24.1)
Discomfort	**–**	67.9 (28.5)	63.9 (29.1)
Waiting	**–**	77.3 (26.5)	77.3 (27.0)
Global index^†^	**–**	73.7 (14.4)	71.7 (14.9)

Furthermore, 96% of patients were willing to learn non-pharmacological approaches to manage anxiety and 87% indicated they would use the COHEC programme again in stressful situations, including 97% for new interventions. All patients were satisfied with the approach to self-manage anxiety and were willing to recommend the COHEC programme. Moreover, 94% of patients were willing to include the COHEC programme in their daily routine.

## Discussion

The results of the study showed that the COHEC programme is feasible, with an optimal adherence rate of 64% in the intention-to-treat population. Although this result falls below the expected adherence, the estimated 95% CI suggests a range of 50–77%. Cardiac coherence practice was optimal: 90.7% of patients achieved cardiac coherence and 88.4% had a 0.1 Hz peak associated with a state of coherence after only 7 days of practice. The included patients were young women without comorbidities undergoing disfiguring BGCSI, a population at higher risk of preoperative anxiety.^2^ Nonetheless, most patients achieved optimal adherence rates, reported feeling calm and relaxed during the exercises, and had completed the COHEC programme at D0. These results are consistent with the literature^13^^,^^21^ and suggest that patients need tools to manage their anxiety, even restrictive ones such as the COHEC programme.

Medical hypnosis practice was heterogeneous among patients, but technical issues limited the access to the medical hypnosis session, especially away from home. Furthermore, 10% of patients reported only partial understanding of the initial training, especially for medical hypnosis. The lack of scheduled medical hypnosis sessions might have hindered treatment adherence.^22^ Moreover, some patients were afraid of hypnosis, including medical hypnosis. Other patients might have been less receptive to medical hypnosis induced by an audiotape.

The COHEC programme enabled the rapid acquisition of skills for autonomous practice. The implementation of plethysmography in clinical practice was rapid, and the results showed good consistency with electrocardiographic measurements. Very few participants (<5%) found the programme complex and were unable to achieve cardiac coherence. These patients might need additional support.^23^ These results suggest that active techniques, such as breathing exercises, might be easier for managing anxiety than listening to medical hypnosis sessions. Both medical hypnosis and cardiac coherence enable disconnecting the executive control network to achieve a state of relaxation and wellbeing by modulating the salience network.^10^^,^^11^ Nevertheless, breathing techniques seem to induce this state more easily. This might explain why hypnotic induction using breathing techniques is faster and more effective in clinical practice.^24^

We aimed to investigate the evolution of anxiety and compared assessment tools as exploratory data in anticipation for a randomised trial to evaluate the efficacy of the COHEC programme. Indeed, anxiety assessment remains challenging, although several tools are available.^25^, ^26^, ^27^, ^28^ In this study, we used VAS and APAIS scales. The VAS scores seemed to remain stable from enrolment to D–1, suggesting that the COHEC programme potentially helped patients to control their anxiety. Additionally, anxiety at levels D–1 (*P*=0.02) and D0 (*P*=0.04) was reduced in patients with severe anxiety (VAS ≥70) at inclusion. Even patients with persistent severe anxiety underwent the intervention without panic attacks. The APAIS total anxiety score seemed in line with VAS results: 52% of the patients with severe anxiety (APAIS total score >11) at inclusion were less anxious (APAIS score <11) at D–1 (*P*=0.01).

In addition, we observed a correlation between VAS and APAIS scores in our cohort, as previously demonstrated.^29^ However, although APAIS total anxiety score distinguishes between severe (score >11) and mild (score <11) anxiety, VAS provides a more detailed assessment of anxiety with at least three levels, especially for patients with moderate to low anxiety. Therefore, VAS seems to be more relevant than APAIS for monitoring therapeutic responses. These results support the use of anxiety as an endpoint for the randomised trial, and the use of VAS to assess it.

Postoperative recovery and patient satisfaction were assessed using the QoR-15 scale, which includes positive and negative dimensions, at D1 and D2, in line with the current practice.^30^ The QoR-15 scores remained stable between inclusion and D1 (*P*=0.70) and increased significantly between inclusion and D2 (*P*=0.04). Some positive dimensions were improved already at D1, particularly nausea and vomiting. The QoR-15 scores were lower than those reported in the literature, but our patients had more severe pathologies and underwent extensive procedures.^31^ The EVAN-G scores showed that patients were satisfied with the COHEC programme. Patients not only followed the COHEC programme but were also willing to learn non-pharmacological approaches to manage their anxiety. Even those who were not initially interested completed the programme. Positive results have also been reported in older patients with psychiatric disorders.^32^

This study has several limitations. The analysis of efficacy endpoints can only be considered exploratory owing to the absence of a control group. Nevertheless, the primary objective was to assess the feasibility of the COHEC programme, and we achieved this objective. Another limitation relates to the study population, which consisted exclusively of women with BGCSI, limiting the generalisability of the results. Women generally experience higher levels of anxiety than men, although this population might be more willing to follow non-pharmacological approaches.^2^ Moreover, adherence to the medical hypnosis sessions was low, primarily because of technical issues that can be easily overcome. Finally, a self-reported questionnaire was used to monitor daily home cardiac coherence practice, which might have biased our results. However, the presence of a peak at 0.1 Hz on the frequency spectrum confirmed the good practice, corroborating our results.

## Conclusions

This study showed the feasibility of the COHEC programme in patients undergoing BGCSI. This programme may potentially help patients to manage preoperative anxiety. A multicentre clinical trial on the COHEC programme funded by the French government is under way to assess the efficacy of the programme (NCT05197972).^33^

## Authors’ contributions

Conceptualisation: JA, AM, CT, MN, LP.

Methodology: JA, MJ, PC, AM, CT, MN, LP.

Investigation including patient enrolment: JA, GL, JD, RG, CD, CV, SRDG, CT, MN.

Clinical data collection and data curation: JA, GL, JD, RG, CD, CV, SRDG, CT, MN, LP.

Statistical analysis: MJ.

Writing–original draft: JA, MJ, PC, AM, LP.

Writing–review: all authors.

Writing–editing: JA, PC, AM, CT, MN, LP.

Project management: PC.

Clinical data monitoring: PC.

All authors read and approved the final version of the manuscript.
